# Is bulk flow plausible in perivascular, paravascular and paravenous channels?

**DOI:** 10.1186/s12987-018-0103-8

**Published:** 2018-06-15

**Authors:** Mohammad M. Faghih, M. Keith Sharp

**Affiliations:** Biofluid Mechanics Laboratory, Department of Mechanical Engineering, University of Louisville, Louisville, KY 40292 USA

**Keywords:** Perivascular flow, Paravascular flow, Paravenous flow, Bulk flow, Brain clearance system, Glymphatic system

## Abstract

**Background:**

Transport of solutes has been observed in the spaces surrounding cerebral arteries and veins. Indeed, transport has been found in opposite directions in two different spaces around arteries. These findings have motivated hypotheses of bulk flow within these spaces. The glymphatic circulation hypothesis involves flow of cerebrospinal fluid from the cortical subarachnoid space to the parenchyma along the paraarterial (extramural, Virchow–Robin) space around arteries, and return flow to the cerebrospinal fluid (CSF) space via paravenous channels. The second hypothesis involves flow of interstitial fluid from the parenchyma to lymphatic vessels along basement membranes between arterial smooth muscle cells.

**Methods:**

This article evaluates the plausibility of steady, pressure-driven flow in these channels with one-dimensional branching models.

**Results:**

According to the models, the hydraulic resistance of arterial basement membranes is too large to accommodate estimated interstitial perfusion of the brain, unless the flow empties to lymphatic ducts after only several generations (still within the parenchyma). The estimated pressure drops required to drive paraarterial and paravenous flows of the same magnitude are not large, but paravenous flow back to the CSF space means that the total pressure difference driving both flows is limited to local pressure differences among the different CSF compartments, which are estimated to be small.

**Conclusions:**

Periarterial flow and glymphatic circulation driven by steady pressure are both found to be implausible, given current estimates of anatomical and fluid dynamic parameters.

## Background

Since the Virchow–Robin space was discovered, there has been disagreement about whether the fluid within is stagnant (as Robin [1] thought) or circulates (opinion held by Virchow [2]) [3]. The recent hypothesis of a “glymphatic” circulation, comprising convection of cerebrospinal fluid from the cortical subarachnoid space to the parenchyma via extramural paraarterial channels and return flow along veins [4], has revived this old question. Further complicating our understanding of flow and transport in this space is evidence of possible flow in the opposite direction within the walls of cerebral arteries, specifically within basement membranes between smooth muscle cell layers (the intramural perivascular space [5]). Motion retrograde to blood flow and to the propagation of the blood pressure pulse is counterintuitive, but a number of models have been developed as possible explanations [6–8]. What has to date not been evaluated, however, is the flow resistance of the full branching paravascular and perivascular networks. Simply put, if the hydraulic resistance of the network exceeds the capability of the available pressure difference to drive significant flow through it, then the steady pressure-driven flow hypothesis is disproven. In this paper, one-dimensional models are developed to test the plausibility of physiologically significant flow in the periarterial, paraarterial and paravenous trees. The anatomy of these spaces is first reviewed in section “Perivascular and paravascular anatomy”, then evidence for solute transport within them and the potential driving mechanisms are outlined in the “Experimental observations of transport and potential mechanisms” section.

### Perivascular and paravascular anatomy

**Fig. 1:**
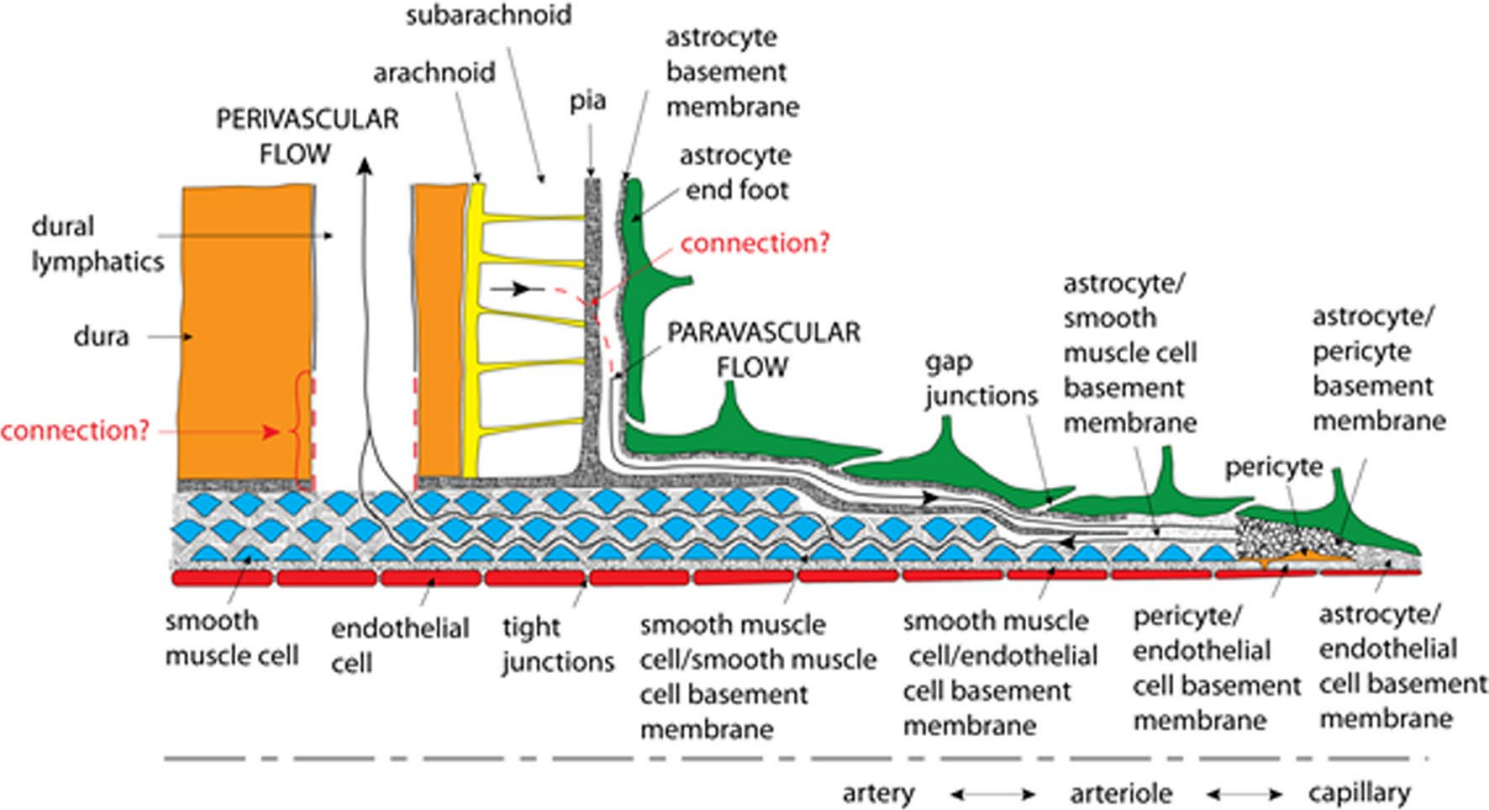
Hypothetical perivascular and paravascular flow pathways in an artery. Paravascular flow moves inward to the brain tissue between astrocyte end feet and pia mater. Perivascular flow moves outward from the brain tissue in basement membranes between SMCs

The anatomy of the perivascular and paravascular channels is shown schematically in Fig. 1. Perivascular describes the basement membranes (about 100 nm thickness [9]) between smooth muscle cells (SMC), which occur in one layer around arterioles, and in 4–20 layers in larger arteries [10].

In the arteries, paravascular refers to the space outside the pia, but inside the astrocyte endfeet forming the glia limitans (Fig. 1). This channel has also been called the Virchow–Robin space [1, 2, 11]. The pial sheath is not found around veins in the parenchyma [12] thus the inner wall of the paravenous space may be the collagen layer between the endothelium and the glia limitans [12]. Interestingly, the space is rapidly and nearly completely closed by cortical spreading depression [13], which may be caused by astrocyte endfoot swelling [14]. This response may have implications for dysfunctions of this clearance pathway and suggests potential for its regulation.

### Experimental observations of transport and potential mechanisms

Transport of molecules with immunological, metabolic and disease-related implications for the brain has been hypothesized in two different directions in the two different channels. First, clearance of amyloid-β suspended in parenchymal interstitial fluid has been hypothesized in the periarterial space [15, 16]. Second, inflow of cerebrospinal fluid from the cortical subarachnoid space to the parenchyma has been hypothesized in the paraarterial space, along with outflow back to the CSF space in the similar gap along cerebral veins (the “glymphatic” system) [17]. The small sizes of these channels make direct measurement of flow challenging, however, the appearance of tracers along the channels has been documented by a number of investigators (e.g., [4, 18]).

While simultaneous flows in opposite directions in the two different channels is theoretically possible [5], two conditions would need to be met. First, a wall with flow resistance greater than that in either channel must exist between the two channels to prevent mixing of the flows. The pia physically separates the two channels in the arteries, but it is unclear whether it has sufficient flow resistance to comprise a hydraulic barrier. Second, the mechanisms driving opposed flows must be identified. Opposed pressure gradients is a candidate mechanism. Since the two channels merge where the pia ends at the precapillaries, the same pressure prevails there. Therefore, opposed flows require pressures higher and lower than that in the precapillary channel in the paravascular and perivascular spaces surrounding the large arteries, respectively. If paraarterial flow originates in the subarachnoid space, and the periarterial flow empties into lymph vessels, then such pressure differences are possible. Paravenous flow back to the CSF space requires that a local pressure difference between CSF compartments, specifically the difference in pressure between the upstream compartment for paraarterial flow and the downstream compartment for paraarterial paravenous flow, is sufficient to drive both flows. The transmantle pressure difference (the difference in pressure between the lateral ventricles and the upper convexity of the subarachnoid space, the largest pressure difference among CSF compartments) is estimated to be no more than 0.03 mmHg [19].

Peristalsis caused by the blood pressure pulse would tend to create flow in the perivascular and paravascular channels in the direction of blood flow. Indeed, Bedussi et al. [20] used a thinned-skull cranial window to image microspheres oscillating at the heart beat frequency and advancing in the direction of blood flow within 20 μm of the surface branches of the middle cerebral artery. However, no evidence was observed of bulk flow into the parenchyma around the penetrating arteries nor clearance around the veins.

Identifying a mechanism for retrograde flow (in the direction opposite that of the blood flow) is essential to validating the periarterial clearance concept. Three hypothesized mechanisms include physical or chemical hinderance of the solute during forward flow, but not during reverse flow [6], flexible flow resistance elements that promote reverse flow [7] and incoherent reflection of waves in the inner and outer walls of the channel [8].

Tracer transport could alternatively be accomplished by molecular diffusion. However, for the relatively large molecules observed in previous experiments, diffusion alone is too slow to explain the rapid spreads observed. Shear-augmented dispersion by oscillatory flow without net bulk flow can increase transport [21]. This possibility was investigated by Sharp et al. [22], but found to be an unlikely explanation for the apparent transport observed in perivascular channels.

Arguably the simplest mechanism for causing bulk flow in the paraarterial space is a steady pressure difference between the subarachnoid space and the parenchyma. This pressure difference is small, about 1 mmHg or less [23, 24]. Two models have been developed of the flow through brain tissue [25, 26], but thus far, none have quantified the relationships between flow and pressure in the channels supplying and emptying the tissue. In this article, the potential for bulk flow within these channels is tested with mathematical models of the periarterial, paraarterial and paravenous trees.

## Methods

### Vascular tree models

In the following subsections, simplified models of periarterial, paraarterial and paravenous trees of annular cross section, through which amyloid-β and other tracers are assumed to flow, are explained.

#### Periarterial

For the periarterial space, the basement membrane between SMC layers was taken as 100 nm thick [9]. This gap between cells forms an irregular path along the vessel, but for simplicity was modeled as an annulus. Depending on the size of the artery, there may be from one layer in precapillaries [27] to 20 layers in large arteries, each forming basement membrane layers between adjacent layers of cells [10]. The hypothesis involves interstitial fluid entering the branching network at the precapillaries and exiting to the lymphatics, thus intracranial pressure prevails upstream and lymphatic pressure downstream.

A one-dimensional analytical solution was obtained that models the flow as steady Poiseuille flow through annular channels with rigid walls. The effect of the porous media in the channels was neglected, as was resistance in the bifurcations. The model consisted of a symmetrical tree from pre-capillaries to the main cerebral arteries.

**Fig. 2:**
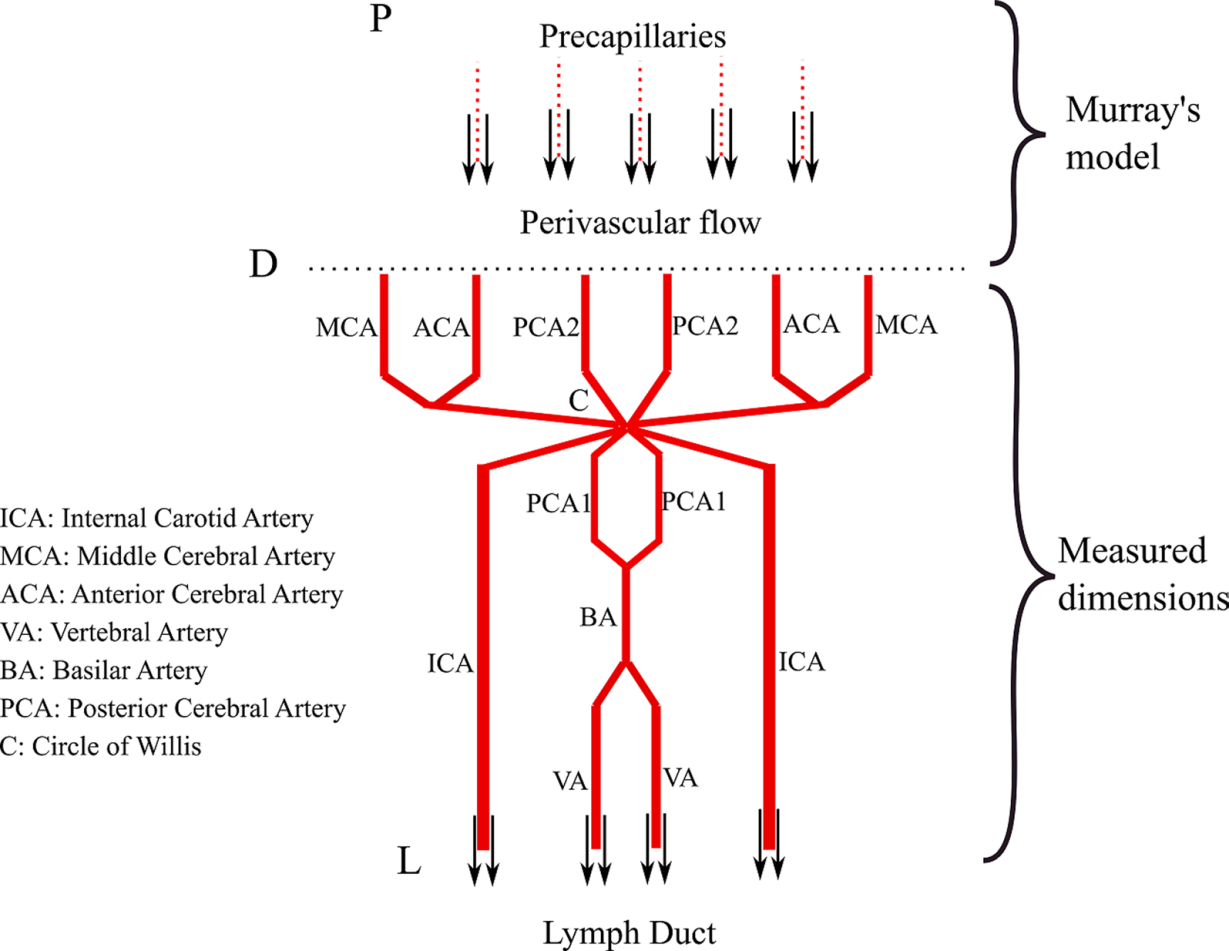
Schematic of the arterial tree

While flow in the periarterial space is hypothesized to be in the opposite direction, the tree model will be described in the more conventional direction of luminal flow. Actual dimensions were used for large arteries (i.e., internal carotid arteries, vertebral artery, basilar artery, anterior, middle and posterior cerebral arteries), for which anatomic data are available (Table 1). The vertebral and internal carotid arteries were connected to the Circle of Willis and then to the middle, anterior and posterior cerebral arteries (Fig. 2). Murray’s law of bifurcations was used to model the bores of the smaller arteries (point D to point P in Fig. 2) [28, 29]. Murray’s law equates the cube of a parent vessel’s diameter to the sum of the cubes of the daughter vessels’ diameters [30]. However, while the exponent in the original Murray’s equation is 3, Cassot et al. [31] showed that the exponent should be modified to 3.67 for human cerebral arteries. The daughter vessels were assumed to have equal diameters. Therefore, the radius of the parent vessel is

1 $$r_{p} = \left( {\frac{1}{2}} \right)^{{\frac{1}{3.67}}} r_{d}$$

where $r_{d}$ is the radius of the daughter vessels. Due to symmetry of the tree, the radius of vessels in a generation can be obtained in terms of the zeroth generation (i.e., largest vessel) by extending Eq. 1 as

2 $$r_{i} = \left( {\frac{1}{2}} \right)^{{\frac{i}{3.67}}} r_{0}\, i = 0, 1, 2, \ldots .$$

**Table 1:** Anatomical sizes of the large arteries (refer to Fig. 2 for definitions of abbreviations) [38, 39]

Artery	Length, mm	Diameter, mm
VA	125	2.5
ICA	142	3.6
BA	28	3.3
PCA1	13	2
PCA2	60	2
MCA	51	3
ACA1	20	2
ACA2	45	2

The vessels MCA, ACA and PCA2 (Fig. 2) were considered to be the zeroth generation (*i *= *0*) of six subtrees. The length of each artery was related to its own radius, which with Eq. 2 is related to that of the zeroth generation [32, 33]

3 $$l_{i} = 20\, r_{i} = 20 \left( {\frac{1}{2}} \right)^{{\frac{i}{3.67}}} r_{0} .$$

Starting from the diameters in Table 1, 30, 28 and 28 generations were required, including the zeroth generation, to reach precapillary diameters of 12.5, 12.2 and 12.2 µm as the final generations in the MCA, ACA and PCA2 subtrees, respectively [10, 34]. (The calculated precapillary diameters are different for each subtree since the zeroth generations have unique diameters.) Including four more generations as capillaries down to 4.7 µm in diameter [35, 36], the total number of capillaries in the model is 98 billion, which agrees with estimates in the literature [37].

The precapillaries, which have only one SMC layer, were nonetheless assumed to each have an annular flow channel of the same gap dimension as one basement membrane. A basement membrane layer was added to each generation of larger arteries up to a maximum of 20 annular channels (at generations 12, 10 and 10 for MCA, ACA and PCA2, respectively). All larger generations were assigned 20 annular channels (21 SMC layers [27]).

Laminar flow resistance for the first annular space (closest to the lumen) at each generation was calculated for Poiseuille flow in an annular cross section [40]

4 $$R = \frac{8 \mu }{{\pi r^{4} }} \left[ {\frac{l}{{\left( {k^{ - 4} - 1} \right) - \frac{{\left( {k^{ - 2} - 1} \right)^{2} }}{{Ln\left( {k^{ - 1} } \right)}}}}} \right]$$

where *R* is the flow resistance, *μ* is the fluid viscosity, *k* = *r*/(*r* + *g*) is the ratio of the inner radius to outer radius, *g* is the gap height of the annulus, and *l* is length of the arterial segment which is related to the radius of the segment by Eq. 3. As mentioned earlier, the thickness of a basement membrane was taken as *g* = 100 nm. For segments with more than one annular cross section, the same relation as Eq. 4 was used to calculate the flow resistance for annular layers other than the first one, with inner radius being *r* + *jg*, where $j = 1,2, \ldots , J$ is the maximum number of annular layers in the generation.

Due to symmetry, the effective resistance of the arterial tree included identical, parallel subtrees representing MCA, ACA and PCA2 pairs.

#### Paraarterial

The model for the paraarterial space starts from the pial arteries (approximately 100 µm in diameter [41, 42]) in the subarachnoid space and ends at the precapillaries. To model this paraarterial part of the glymphatic system, the periarterial model was modified with different starting locations and annular spaces with different gaps. The modified model began at generations 18, 16 and 16 for MCA, ACA and PCA2 branches, respectively, where artery diameters were 100.16, 97.42 and 97.42 µm, respectively. The ratio of outer paraarterial radius to the lumen radius was assumed to be constant through the tree and equal to 1.12 [13] (about 12 µm gaps for the largest arteries of all three branches), except in the precapillaries where the annular gap was again assumed to be *g* = 100 nm [20]. Using this ratio (i.e., 1.12), the ratio of inner radius to outer radius in the paraarterial tree was calculated to be *k* = 0.6652. Flow resistance in each branch was calculated using Eq. 4.

#### Paravenous

The paravenous space begins at the postcapillaries just after the capillaries. The number of postcapillaries was taken to be the same as the number of precapillaries [34], but the diameter (20 μm) of postcapillaries was slightly larger [34, 43]. Taking the power in Murray’s law as 3.54 for veins [31], after 10 generations the diameter of pial veins became 141.7 µm, which is in approximate agreement with observations [44, 45]. Equation 3 was again assumed to scale the length of veins, and Eq. 4 was used to calculate the flow resistance for the paravenous tree, except that *k* = 0.94, based on the ratio of paravenous to luminal area of 0.13 found for veins [13] (about a 18 µm gap for the pial veins).

### Case conditions

The density and kinematic viscosity of interstitial and cerebrospinal fluid taken to be that of water at body temperature, ρ = 993 kg/m^3^ and ν = 7 × 10^−7^ m^2^/s.

The resistance of the perivascular model was used to calculate the interstitial fluid perfusion that would result from a pressure drop of 14 mmHg, representing a typical difference between intracranial and lymphatic duct pressures [46]. These flow rates were compared to two different estimates of interstitial fluid perfusion. First, extrapolating from estimated interstitial fluid production in the rat brain of 0.1–0.3 µl/min/g [47, 48], flow rates in the human brain become 0.13–0.39 ml/min (assuming a mass of 1.3 kg). Second, since the brain receives about 15% of the total cardiac output [49], another estimate can be calculated as 15% of the lymphatic flow rate in the whole body of 1.4–2.1 ml/min [50, 51], which gives 0.21–0.32 ml/min. These estimates are in substantial agreement.

For the paraarterial model, the pressure difference necessary to drive the minimum flow rate of 0.13 ml/min from the cortical subarachnoid space to the parenchyma (and from parenchyma to CSF space for the paravenous model) was calculated.

## Results

In this section, results of flow resistance for the periarterial, paraarterial and paravenous tree models, described above, are presented.

### Periarterial flow

**Fig. 3:**
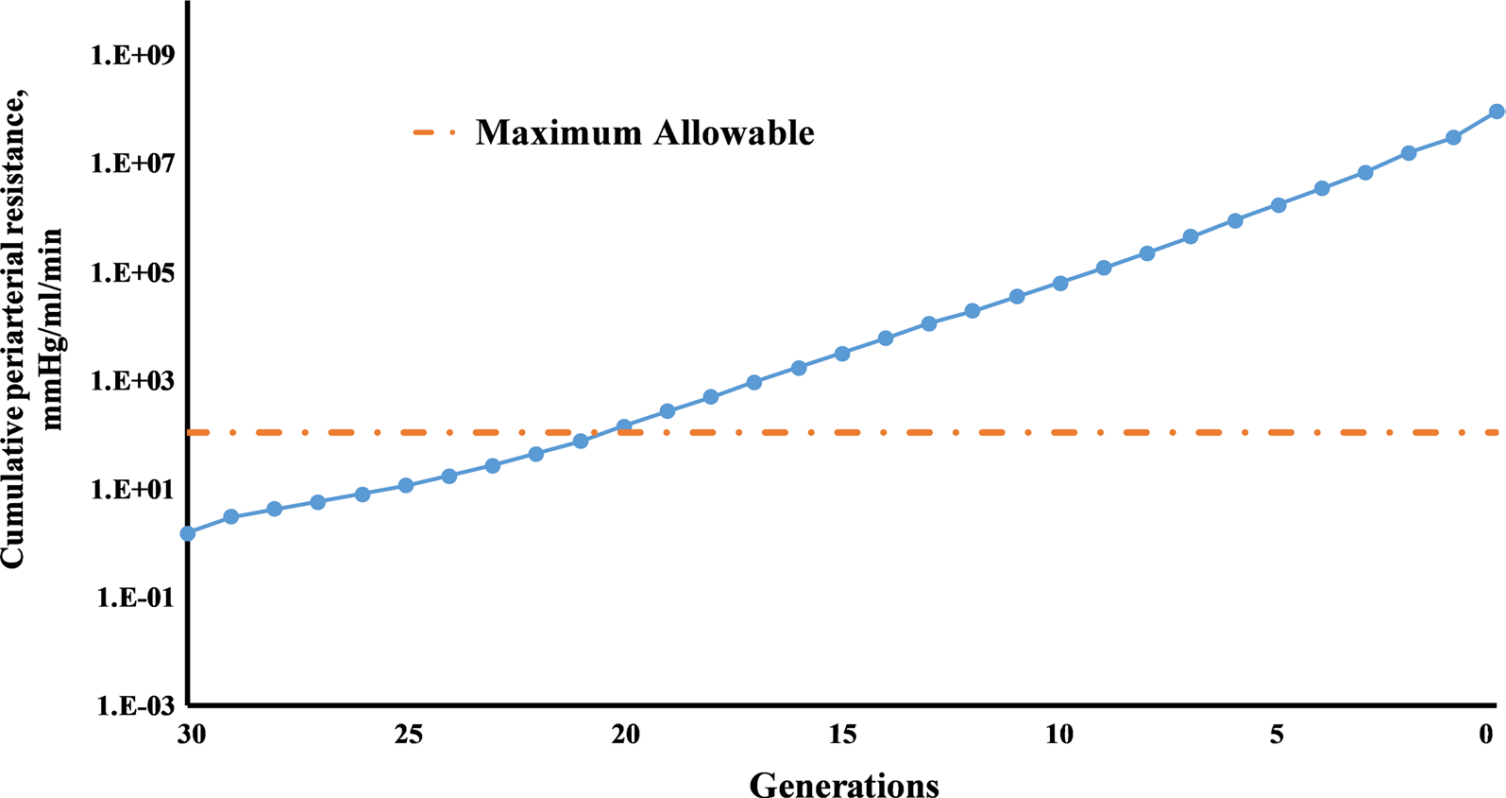
Cumulative periarterial resistance

Periarterial resistance of the large arteries upstream of the Circle of Willis (between points L and C in Fig. 2) was calculated to be 2.13 × 10^8^ mmHg/ml/min. Periarterial resistance from the Circle of Willis to the precapillaries (between points C and P) was equal to 1.4 × 10^8^ mmHg/ml/min. Therefore, the total periarterial flow resistance is the sum of these two values, 3.53 × 10^8^ mmHg/ml/min (the full cumulative resistance at the zeroth generation in Fig. 3).

For comparison, taking the typical pressure difference of 14 mmHg between the parenchyma and lymphatic ducts and the lower limit of the range of interstitial fluid production of 0.13 ml/min gives a maximum flow resistance of 107.76 mmHg/ml/min to allow physiologic interstitial fluid clearance by the periarterial pathway (the dashed line in Fig. 3). To not exceed this maximum resistance, the flow would need to exit the periarterial tree to lymphatic ducts after no more than 10 generations (generations 30–21, Fig. 3). The diameters of the 21st generations are 56.83, 37.89 and 37.89 µm for MCA, ACA and PCA2 branches, which is still 3, 5 and 5 generations away from the pial arteries, respectively.

### Paraarterial flow

**Fig. 4:**
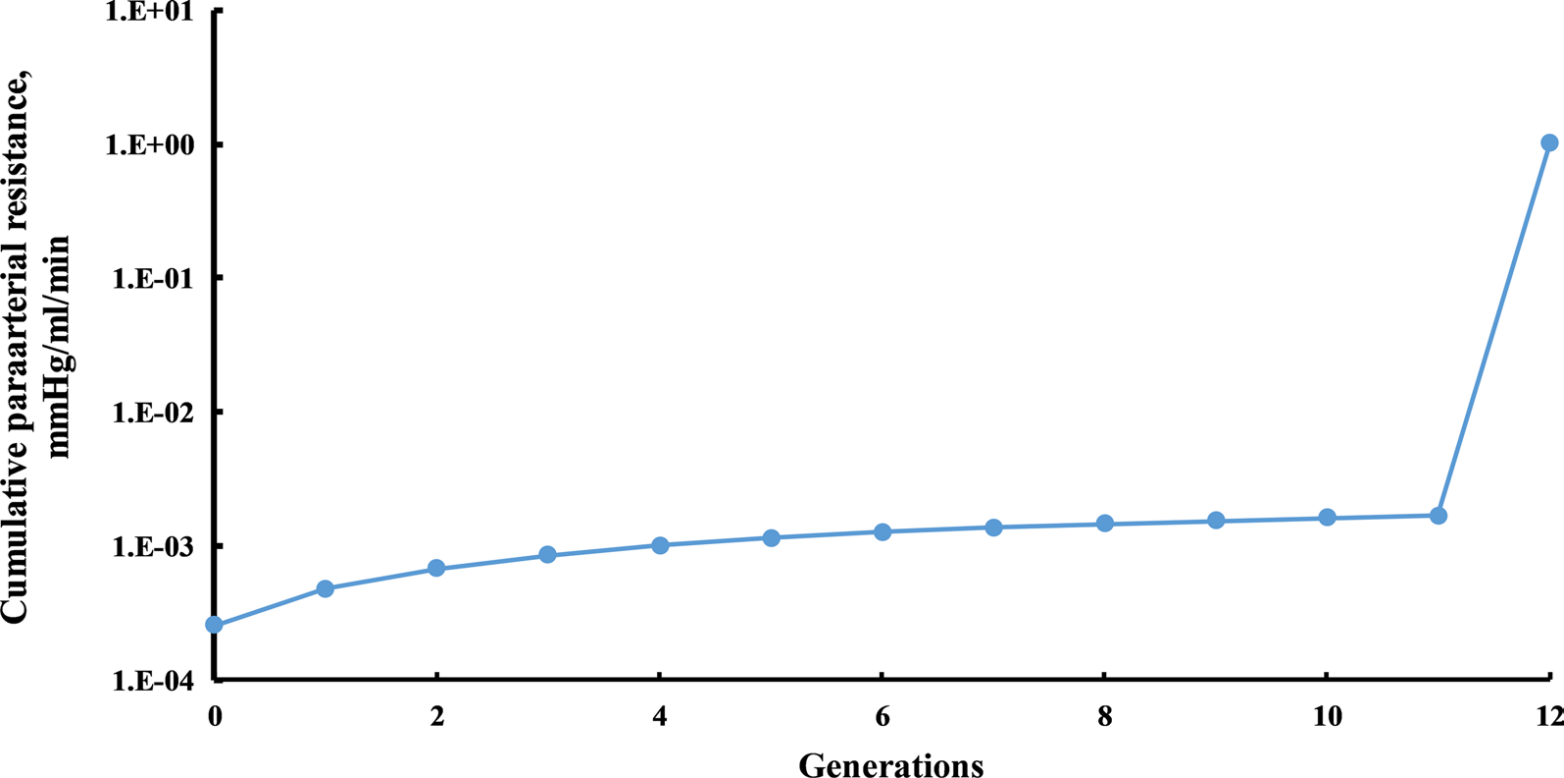
Cumulative paraarterial resistance

The total resistance of the paraarterial model was calculated to be 1.14 mmHg/ml/min (Fig. 4). As can be seen in Fig. 4, the resistance of the paraarterial tree model is dominated by the small gaps in the precapillaries. If flow in the tree exits to the parenchyma earlier, then the resistance is about three orders of magnitude lower. Since the glymphatic circulation in the paraarterial space is hypothesized to originate in the cortical subarachnoid space and terminate in the parenchyma, a large pressure difference between the two ends is not expected. Therefore, the approach taken was to calculate the pressure difference required to cause the lowest flow rate of 0.13 ml/min through the paraarterial tree. This lowest required pressure difference was 0.15 mmHg.

### Paravenous flow

**Fig. 5:**
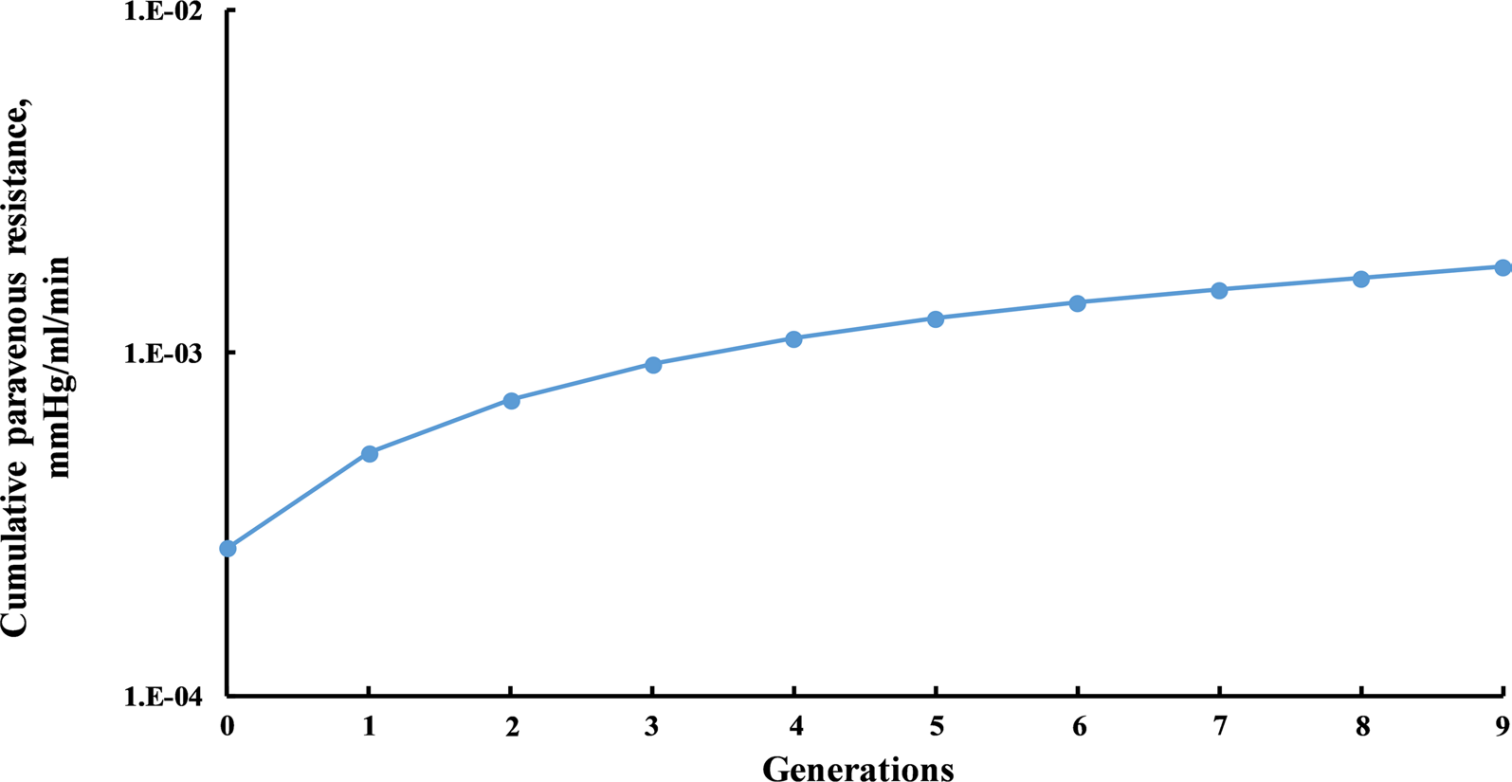
Cumulative paravenous resistance

The total resistance of the paravenous tree was equal to 1.75 × 10^−3^ mmHg/ml/min, about three orders of magnitude smaller than that of the paraarterial tree (Fig. 5), which can be expected based on the larger gaps and larger vessel diameters compared to the paraarterial channels. A more consistent generation-to-generation increase in resistance is also evident. The required pressure difference to drive 0.13 ml/min of flow through the paravenous tree was calculated to be 0.00023 mmHg. If flow entered from the parenchyma later than the post capillaries, resistance would be even lower.

## Discussion

The resistance of the full periarterial tree is approximately 4 million times too large to be a plausible pathway for steady, pressure-driven clearance. For 14 mmHg of pressure to drive 0.13 ml/min of flow, the periarterial tree would have to terminate at the 21st generation, which is still within the parenchyma.

Only 0.15 mmHg of pressure between the cortical subarachnoid space and the parenchyma is required to drive the same flow through the larger (larger annular gap) and shorter paraarterial tree. Such a pressure difference is not implausible, since it is within the range of estimates for this pressure difference [23, 24]. However, the hypothesized paravenous flow also terminates in the CSF space. Therefore, the total pressure difference driving both paraarterial and paravenous flows can be no greater than the transmantle pressure, which is estimated to be no greater than 0.03 mmHg [19]. The required paraarterial pressure difference alone being larger than this means that combined steady pressure-driven glymphatic flow along the entire length of both trees is unlikely.

If, however, flow exits the paraarterial tree before the precapillaries, the cumulative resistance of the paraarterial tree is 1.68 × 10^−3^ mmHg/ml/min. In this case, the pressure difference required to drive 0.13 ml/min of flow through both trees is 0.00045 mmHg, which is considerably less than the maximum transmantle pressure.

Because the cranium has low compliance, injections increase pressure in the space in which they occur. For instance, Iliff et al. [52] reported a 2.5 mmHg elevation of intracranial pressure during a 10 μl injection of tracer at a rate of 1 μl/min into the cisterna magna. According to the models in this work, this increase in pressure is significantly larger than that required to drive flow in the paravascular spaces. While some investigators have used smaller injection rates (e.g., Carare et al. [18] used injections of 0.5 μl over at least 2 min), observed transport may be in part an artifact of the location of injection.

On the other hand, the evidence for flow in these spaces is based on observation of the appearance of tracers in the channels some time after injection into the cerebrospinal fluid space or parenchyma. Therefore, solute, but not solvent, transport is a less stringent requirement to explain these observations. Shear-augmented dispersion [22] and streaming [53] are possible mechanisms that can cause tracer transport in the absence of net bulk flow in a particular direction.

Limitations of the models include ignoring the tortuosity of the channels and the effects of branches and porous media, all of which would increase resistance, making it more difficult to explain hydraulically-driven flow in these channels.

A Darcy–Brinkman model can be used to estimate the influence of porous media. Using this model, the increase in resistance of the channel for large Darcy number *Da* scales with *Da*^2^/3 [54]. For basement membranes with permeability of 1.432 × 10^−18^ m^2^ [55], *Da* becomes 41.8 and resistance in the periarterial channels with porous media is 582 times higher than without porous media. The increase in resistance in the paravascular spaces depends on the gap dimension, with the largest increase occurring for the largest gap (surrounding the largest vessels). For a 12 µm gap around the largest arteries of the paraarterial tree and with an estimated permeability of 1.8 × 10^−14^ m^2^ [56], *Da* becomes 44.7 and resistance in the largest paraarterial channels with porous media is 667 times higher. For a 18.4 µm gap around the pial veins of the paravenous tree and with the same estimated permeability, *Da* becomes 67.6 and resistance in the largest paravenous channels with porous media is 1567 times higher.

With porous media, the resistance of the periarterial tree becomes about 2 billion times too large to support the estimated physiologic flow. This result further reinforces the implausibility of pressure-driven flow in these channels.

Applying the resistance increases due to porous media estimated above to the entire paravascular trees, the required pressure differences become 99 and 0.36 mmHg for the paraarterial and paravenous trees, respectively. The necessary paravenous pressure difference is still small. The required paraarterial pressure difference, however, is beyond the range measured or theorized between the parenchyma and CSF spaces. To be limited to the transmantle pressure, flow would need to exit the pararterial tree earlier and enter the paravenous tree later. The total resistance of the two truncated trees could be no larger than 0.23 mmHg/ml/min for the transmantle pressure to drive 0.13 ml/min of flow. Maximum truncation would correspond to pial arteries only for the paraarterial tree and pial veins only for the paravenous tree. Without porous media, the resistances of the paraarterial channels surrounding the pial arteries and the paravenous channels surrounding the pial veins are 2.56 × 10^−4^ and 2.69 × 10^−4^ mmHg/ml/min, respectively (Figs. 4 and 5). With the Darcy numbers estimated above, the resistance of the paraarterial channels becomes 0.171 mmHg/ml/min, and that of the paravenous channels becomes 0.422 mmHg/ml/min. The combined resistance exceeds the transmantle pressure by a factor of 19.7. Though this rather large factor suggests that significant glymphatic circulation does not occur, the uncertainties of the accuracy of anatomical and kinematic variables involved in these estimates dictate caution regarding such a conclusion. If five estimates were in error by factors of 1.8 (say, roughly half the flow rate driven by twice the transmantle pressure in twice as many vessels with double the gap and double the permeability), then agreement would be obtained. This possibility highlights the need for in vivo measurements of these parameters.

Peristalsis represents an alternative mechanism for driving flows in these channels. The maximum peristaltic pressure that could possibly occur in the channels surrounding arteries can be estimated as the carotid artery pulse pressure of about 40 mmHg. This pressure is substantially higher than the 14 mmHg available for retrograde periarterial flow and the 0.03 mmHg transmantle pressure for paravascular flows. However, a confounding factor is that the wavelength of the blood pressure pulse (~ 10 m [57]) is much longer than the cerebral vessels. Under these conditions, arterial wall motion occurs nearly simultaneously along the entire channel, thus axial pressure gradients and the cycle-averaged flow in a particular direction that can be drive by them are small [25, 26]. Other contributing mechanisms in combination with wall motion are necessary to drive significant flow. (See, for instance, [6–8]. While the focus of these papers are on explaining retrograde flow in the periarterial space, similar, reversed mechanisms could promote forward flow in the paraarterial space.) Because venous pressure is less pulsatile, the potential for peristaltically-driven flow in the paravenous space is lower. With porous media, however, the estimated necessary pressure difference of 99 mmHg is double that available from the arterial pulse pressure. The additional resistance of porous media makes peristalsis a questionable driver of paraarterial flows even if another mechanism promotes forward flow.

## Conclusions

Significant steady pressure-driven flow in the periarterial space is found to be unlikely, unless flow exits to the lymphatic circulation after only a few generations. An outlet to the lymphatic system at this early level has not been identified. With channel resistance increased by two orders of magnitude by porous media, steady pressure-driven flow becomes even less plausible.

A fundamental paradox of the glymphatic circulation is that cortical subarachnoid space pressure must be high to drive steady flow through paraarterial channels, but low pressure must prevail in the CSF space terminus downstream of the paravenous channels to draw flow through these channels. Even without porous media, the combined pressure difference required to drive flow through both trees exceeds the maximum transmantle pressure. With porous media, the necessary pressure is at least two orders of magnitude higher. Therefore, steady pressure-driven glymphatic flow through the entirety of both trees is also implausible. Predictions are less clear for flow through truncated trees. With porous media, the combined resistance of the paravascular spaces of just the pial arteries and veins also exceeds the transmantle pressure. However, the mismatch is small enough that uncertainties in parameter estimates limit confidence in a conclusion of implausibility of flow.

Although the blood pressure pulse wavelength is too long to allow peristalsis alone to drive these flows, the current results cannot rule out its importance in combination with another mechanism [6–8]. So far, these contributing mechanisms have not been confirmed by experiments, nor have the models been applied to branching networks of channels to determine the magnitude of total brain perfusion that could result. Both avenues of further investigation could yield valuable insights to explain the transport of tracers observed in experiments.

## Abbreviations

ACA: anterior cerebral artery
MCA: middle cerebral artery
PCA: posterior cerebral artery
SMC: smooth muscle cells
